# Remodeling Pattern of Spinal Canal after Full Endoscopic Uniportal Lumbar Endoscopic Unilateral Laminotomy for Bilateral Decompression: One Year Repetitive MRI and Clinical Follow-Up Evaluation

**DOI:** 10.3390/diagnostics12040793

**Published:** 2022-03-24

**Authors:** Hyeun-Sung Kim, Pang-Hung Wu, Giovanni Grasso, Jin-Woo An, Myeonghun Kim, Inkyung Lee, Jong-Seon Park, Jun-Hyoung Lee, Sangsoo Kang, Jeongshik Lee, Yeonjin Yi, Jun-Hyung Lee, Jun-Hwan Park, Jae-Hyeon Lim, Il-Tae Jang

**Affiliations:** 1 Spine Surgery, Nanoori Gangnam Hospital, Seoul 06048, Korea; 2 Department of Orthopaedic Surgery, Juronghealth Campus, National University Health System, Singapore 609606, Singapore; 3 Neurosurgical Clinic, Department of Biomedicine, Neurosciences and Advanced Diagnostics University for Palermo, 90127 Palermo, Italy; giovanni.grasso@unipa.it; 4 Nanoori Spine and Joint Clinic with Saudi German Hospital in Dubai, Dubai 66566, United Arab Emirates; drbear94@gmail.com; 5 Department of Neurosurgery, Nanoori Hospital Gangnam, Seoul 06048, Korea; michaelkmh@hanmail.net (M.K.); ikl84@naver.com (I.L.); siren7317@naver.com (J.-S.P.); drbrainlee@gmail.com (J.-H.L.); ellinore@gnnanoori.co.kr (S.K.); nsjslee@hanmail.net (J.L.); nigaheboa@hanmail.net (Y.Y.); pp3614@naver.com (J.-H.L.); nanoolim@naver.com (J.-H.L.); nanoori_research@naver.co.kr (I.-T.J.); 6 The Faculty of Medicine, University of Debrecen, Nagyerdei krt. 94, 4032 Debrecen, Hungary; yyea7133@gmail.com

**Keywords:** endoscopic spine surgery, lumbar endoscopic unilateral laminotomy with bilateral decompression, degenerative spine disease, spinal stenosis, remodeling of spine, minimally invasive spine surgery

## Abstract

Objective: There is limited literature on repetitive postoperative MRI and clinical evaluation after Uniportal Lumbar Endoscopic Unilateral Laminotomy for Bilateral Decompression. Methods: Clinical visual analog scale, Oswestry Disability Index, McNab’s criteria evaluation and MRI evaluation of the axial cut spinal canal area of the upper end plate, mid disc and lower end plate were performed for patients who underwent single-level Uniportal Lumbar Endoscopic Unilateral Laminotomy for Bilateral Decompression. From the evaluation of the axial cut MRI, four types of patterns of remodeling were identified: type A: continuous expanded spinal canal, type B: restenosis with delayed expansion, type C: progressive expansion and type D: restenosis. Result: A total of 126 patients with single-level Uniportal Lumbar Endoscopic Unilateral Laminotomy for Bilateral Decompression were recruited with a minimum follow-up of 26 months. Thirty-six type A, fifty type B, thirty type C and ten type D patterns of spinal canal remodeling were observed. All four types of patterns of remodeling had statistically significant improvement in VAS at final follow-up compared to the preoperative state with type A (5.59 ± 1.58), B (5.58 ± 1.71), C (5.58 ± 1.71) and D (5.27 ± 1.68), *p* < 0.05. ODI was significantly improved at final follow-up with type A (49.19 ± 10.51), B (50.00 ± 11.29), C (45.60 ± 10.58) and D (45.60 ± 10.58), *p* < 0.05. A significant MRI axial cut increment of the spinal canal area was found at the upper endplate at postoperative day one and one year with type A (39.16 ± 22.73; 28.00 ± 42.57) mm^2^, B (47.42 ± 18.77; 42.38 ± 19.29) mm^2^, C (51.45 ± 18.16; 49.49 ± 18.41) mm^2^ and D (49.10 ± 23.05; 38.18 ± 18.94) mm^2^, respectively, *p* < 0.05. Similar significant increment was found at the mid-disc at postoperative day one, 6 months and one year with type A (55.16 ± 27.51; 37.23 ± 25.88; 44.86 ± 25.73) mm^2^, B (72.83 ± 23.87; 49.79 ± 21.93; 62.94 ± 24.43) mm^2^, C (66.85 ± 34.48; 54.92 ± 30.70; 64.33 ± 31.82) mm^2^ and D (71.65 ± 16.87; 41.55 ± 12.92; 49.83 ± 13.31) mm^2^ and the lower endplate at postoperative day one and one year with type A (49.89 ± 34.50; 41.04 ± 28.56) mm^2^, B (63.63 ± 23.70; 54.72 ± 24.29) mm^2^, C (58.50 ± 24.27; 55.32 ± 22.49) mm^2^ and D (81.43 ± 16.81; 58.40 ± 18.05) mm^2^ at postoperative day one and one year, respectively, *p* < 0.05. Conclusions: After full endoscopic lumbar decompression, despite achieving sufficient decompression immediately postoperatively, varying severity of asymptomatic restenosis was found in postoperative six months MRI without clinical significance. Further remodeling with a varying degree of increment of the spinal canal area occurs at postoperative one year with overall good clinical outcomes.

## 1. Introduction

Spinal stenosis is the leading cause of claudication in aging populations. Increased prevalence of spinal stenosis leads to a similar increment in spinal decompressive surgeries performed. This leads to strong demand for minimally invasive surgery such as endoscopic surgery. The evolution of endoscopic spine surgery equipment and techniques allows more complex lumbar procedures to be performed using spinal endoscopy [1,2,3]. Endoscopic approaches through the transforaminal and interlaminar route in lumbar decompression and discectomy have been well described [4,5,6,7,8]. Several authors described in detail the step-by-step approach to performing lumbar endoscopic unilateral laminotomy with bilateral decompression (LE-ULBD) [9,10,11,12]. Kim and Wu et al. described 12 key steps in LE-ULBD which consist of (1) preoperative preparation; (2) skin incision over the laminofacet junction, the “V” point; (3) docking of the endoscope at the “V” point; (4) ipsilateral partial inferior and superior articular facet resection; (5) ipsilateral cephalad laminotomy; (6) base of spinous process resection; (7) ipsilateral caudal laminotomy; (8) contralateral sublaminar cephalad laminotomy; (9) contralateral caudal laminotomy; (10) contralateral partial superior articular facet resection; (11) flavectomy; (12) hemostasis and final checking of decompression of neural elements [9]. There is evidence of good clinical outcomes in patients who underwent lumbar endoscopic unilateral laminotomy with bilateral decompression [9,10,11,12]. Remodeling of ligamentum flavum after lumbar interbody fusion has been evaluated in the literature [13,14]. There are limited studies of the postoperative remodeling process of patients who underwent lumbar endoscopic unilateral laminotomy with bilateral decompression. The objective of our study is to evaluate the clinical and radiological parameters in a cohort of patients who underwent unilateral laminotomy with bilateral decompression. 

## 2. Materials and Methods

### 2.1. Indication, Inclusion and Exclusion Criteria

Informed consent was obtained from all patients who participated in this retrospective comparative study, which was reviewed by the institutional review board of Nanoori Hospital, Seoul, Republic of Korea (NR-IRB 2021-009). 

The patients included in the study presented with neurogenic claudication with failure of a minimum of 6 weeks of conservative treatment. They presented with either one or more of the following findings: (1) neurogenic claudication (2) grade 1 spondylolisthesis or no spondylolisthesis seen on flexion and extension X-rays (3) MRI demonstrated spinal stenosis. Neurogenic claudication was found in patients with mechanical radicular leg pain starting in the lumbosacral region radiating to the dermatomal region in concordance with an MRI showing spinal stenosis relieved by flexion posture or sitting exacerbated by walking with normal pulses felt in bilateral feet. Each patient had a single level uniportal lumbar endoscopic unilateral laminotomy with bilateral decompression (LE-ULBD) with or without interlaminar contralateral endoscopic lumbar foraminotomy (ICELF). Additional ICELF was indicated in patients who had foraminal stenosis in addition to central and lateral recess stenosis. We evaluate the clinical and radiological outcomes of this cohort of patients. 

We excluded patients who had vascular claudication, revision spinal surgery, trauma, tumour, pseduoarthrosis, infection, congenital spinal deformity, sagittal malalignment and coronal malalignment with more than 10 degrees coronal curve. 

### 2.2. Surgical Technique of Lumbar Endoscopic Unilateral Laminotomy with Bilateral Decompression and Interlaminar Contralateral Endoscopic Lumbar Foraminotomy 

**Figure 1:**
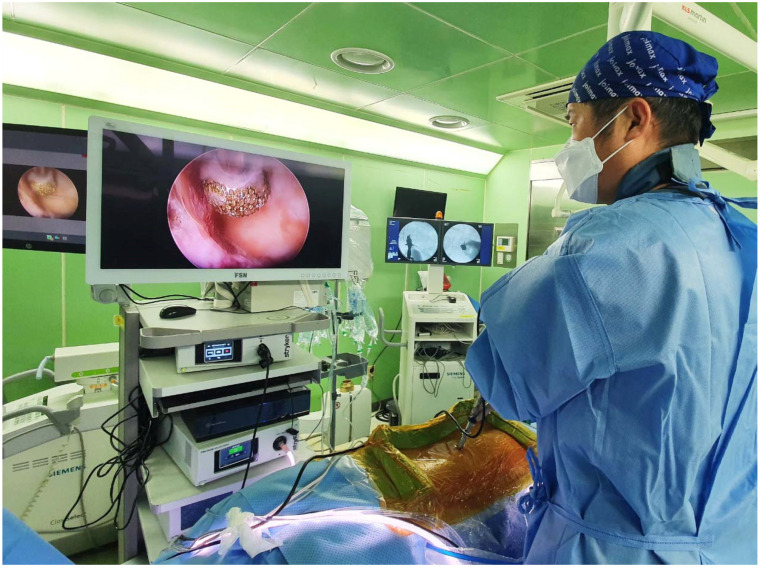
Intraoperative picture shows the set up for lumbar endoscopic unilateral laminotomy with bilateral decompression (LE-ULBD). The patient lies in a prone position with the operating surgeon standing on the same side as the lesion of the lumbar segment. The endoscopic monitor tower is placed directly opposite the surgeon with fluoroscopic equipment adjacent to the endoscopic monitor tower.

**Figure 2:**
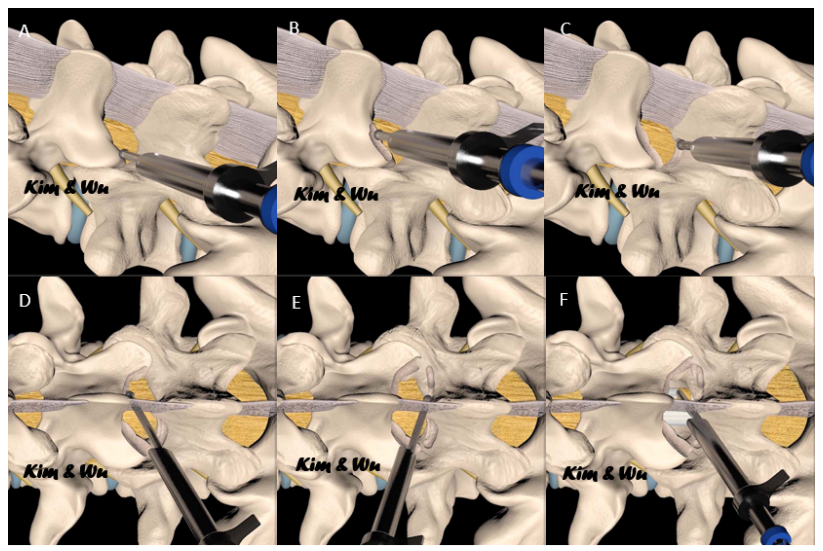
Graphical sketch of the steps of lumbar endoscopic unilateral laminotomy with bilateral decompression (LE-ULBD). (**A**) endoscopic decompression of the ipsilateral facet joint. (**B**) decompression continues to the ipsilateral lamina, (**C**) ipsilateral caudal lamina decompression is performed. (**D**) After decompression of base of the spinous process of the cephalad lamina, the contralateral decompression of the cephalad lamina is performed over the top of the ligamentum flavum (**E**) after decompression of the base of the spinous process of the caudal lamina, the contralateral decompression of the caudal lamina is performed over the top of the ligamentum flavum. (**F**) Ligamentum flavum removed after completion of endoscopic decompression.

Kim and Wu et al. described an outside-in approach of LE-ULBD [9,15,16] and ICELF [4,17,18]. This surgical technique was adopted in this cohort of patients. A summary of the key steps is discussed here. In LE-ULBD, the surgeon stands and makes a skin incision on the same side as the leg pain or the side with more radiological signs of spinal stenosis if there were bilateral leg pains (Figure 1). A 1–1.5 cm incision and serial dilation was made with a beveled 13 mm outer diameter working cannula docked on at the laminofacet junction, or “V” point. We used a stenosis scope with a 15° viewing angle, an outer diameter of 10 mm, a working channel diameter of 6 mm, and a working length 125-mm endoscope. Endoscopic diamond drills of 3.0–4.5 mm in diameter were used in bony decompression. Bony decompression was performed in the sequence of (1) ipsilateral inferior articular facet, (2) ipsilateral cephalad lamina, (3) ipsilateral superior articular facet, (4) ipsilateral caudal lamina, (5) contralateral cephalad lamina, (6) contralateral inferior articular process, (7) contralateral caudal lamina and (8) contralateral superior articular process. Once bony decompression was satisfactory, ligamentum flava were removed with forceps and endoscopic Kerisson’s rongeurs. Additional ICELF procedure was performed in patients with contralateral foraminal stenosis. Upon completion of LE-ULBD, we switched to a transforaminal endoscope with a 30°, 7.3-mm-outer diameter and 171-mm-length endoscope docked at the contralateral superior articular facet ventral and distal to the contralateral exiting nerve root. We performed foraminal decompression from inside-out starting on the medial aspect of the superior articular process to the lateral margin of the superior articular process. Contralateral end plate syndesmophytes, disc protrusions, superior articular process osteophytes and foraminal ligaments were decompressed (Figure 2).

### 2.3. Collection of Operative, Clinical and Radiological Data

The cohort of patients underwent single-level LE-ULBD performed in the period September 2018 to December 2019. 

We collected and analyzed baseline demographic data. Preoperative and postoperative radiographic magnetic resonance imaging axial cut spinal canal area (SCA) in upper endplate, mid-disc and lower endplate were collected at preoperative, immediate post-operative day one, 6 months and one year. We measured clinical outcomes of the Visual Analogue Scale (VAS) and Oswestry Disability Index (ODI) at preoperative, 1 week postoperative, 3 months postoperative and final follow-up. MacNab’s criteria were evaluated at final follow-up. X-ray was performed preoperatively, and postoperative day one and final follow-up. 

### 2.4. Statistical Analysis

Clinical data were analyzed with SPSS version 18 statistical analysis software (IBM Corporation, New York). The continuous variables were expressed as mean and standard deviation (SD). The *paired t*-test was used for comparison of pre-operative and post-operative radiological MRI results on SCA. Clinical parameters VAS and ODI were analyzed with paired *t*-test. A value of (*p* < 0.05) was considered significant within each group of data. An independent *t*-test was used to compare the clinical data of VAS and ODI and MRI results between the subgroup of type A to D

## 3. Results

### 3.1. Baseline Demographics

**Figure 3:**
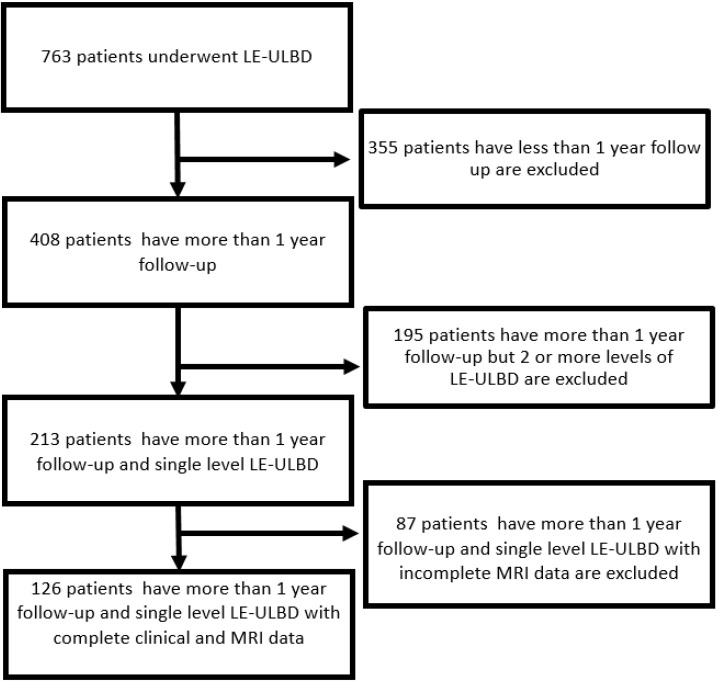
Flowchart of the number of included and excluded patients.

From the period of February 2018 to December 2019, 408 patients who had undergone single or multiple levels of lumbar endoscopic unilateral laminotomy with bilateral decompression (LE-ULBD) were found from our database. A total of 213 patients underwent single-level LE-ULBD with more than one year of follow-up. Eighty-seven patients who did not perform postoperative MRI at 6 months or one year were excluded. A total of 126 patients who underwent LE-ULBD with postoperative MRI at six months and one year with complete clinical data at one-year follow-up met the inclusion and exclusion criteria (Figure 3). The mean age of patients was 63.8 (21–86), with a mean follow-up of 27.6 (17–38) months. 

During the analysis of radiological data, we found that all LE-ULBD stenosis showed significant improvement in SCA at postoperative day one, a variable extent of decrease in the improved SCA by postoperative 6 months and subsequently a variable degree of remodeling by postoperative one year. To analyze these patterns of remodeling, four types of MRI SCA patterns of remodeling were identified: type A: continuous expanded spinal canal, type B: restenosis with delayed expansion, type C: progressive expansion and type D: restenosis. 

In all types, postoperative day one, MRI showed expansion of SCA.

**Figure 4:**
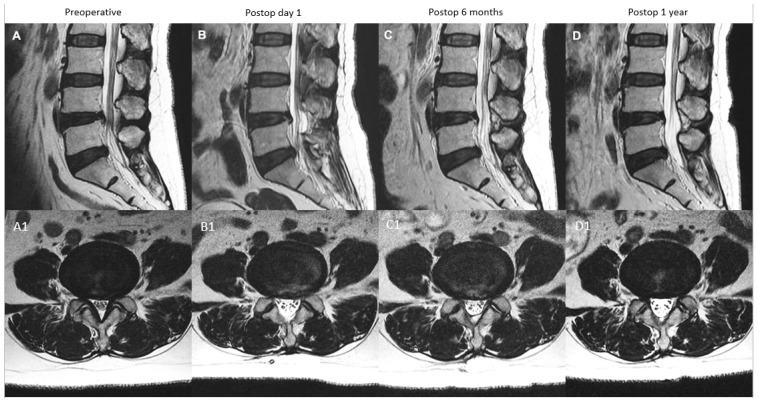
MRI appearance of remodeling patterns at preoperative, postoperative day 1, postoperative 6 months and postoperative 1 year with mid-sagittal cut in the top row and corresponding axial cut in the bottom row. Type A: continuous type, preoperative spinal stenosis at L4/5 (**A**,**A1**) with increased spinal canal area on postoperative day one MRI (**B**,**B1**). There was modest restenosis that occurred at postoperative 6 months (**C**,**C1**), which was maintained at postoperative one year with overall improved spinal canal area (**D**,**D1**).

In type A (continuous expanded spinal canal): SCA expanded significantly on postoperative day one to maximal. Postoperative 6 months SCA measured ≤30% decrease in SCA expanded state at postoperative 6 months compared to postoperative day one. SCA measured at one year was similar to postoperative 6 months, which showed a 10–30% decrease from postoperative day one SCA (Figure 4). 

**Figure 5:**
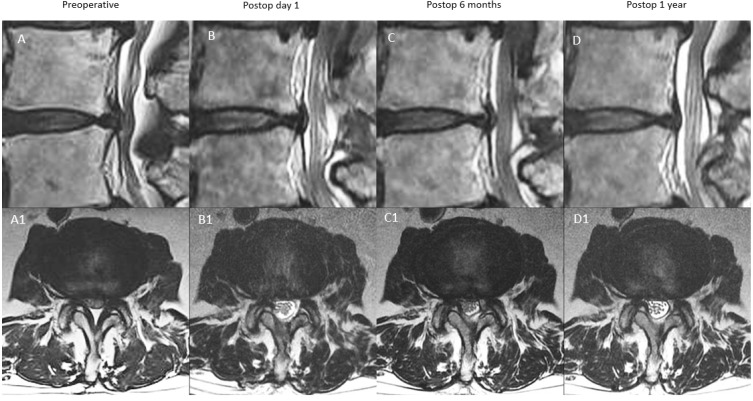
MRI appearance of remodeling patterns at preoperative, postoperative day 1, postoperative 6 months and postoperative 1 year with mid-sagittal cut in the top row and corresponding axial cut in the bottom row. type B: remodeling type, preoperative spinal stenosis of L3/4 (**A**,**A1**) with increased spinal canal area on postoperative day one MRI (**B**,**B1**). There was moderate restenosis that occurred at postoperative 6 months (**C**,**C1**), which improved modestly at postoperative one year with overall improved spinal canal area (**D**,**D1**).

In type B (remodeling with restenosis with delayed expansion): SCA expanded on postoperative day 1. Postoperative 6 months SCA measured >30% decrease compared to postoperative day 1 SCA. Postoperative 1 year SCA measurement improved compared to postoperative 6 months with final SCA less than or equal to 30% of postoperative day 1 SCA (Figure 5). 

**Figure 6:**
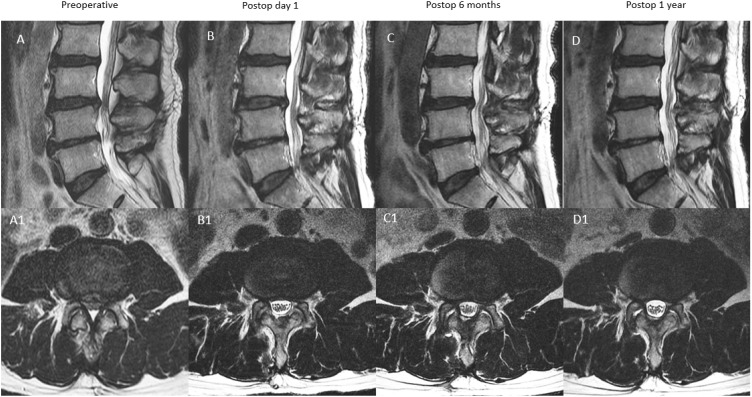
MRI appearance of remodeling patterns at preoperative, postoperative day 1, postoperative 6 months and postoperative 1 year with mid-sagittal cut in the top row and corresponding axial cut in the bottom row. Type C: expansion type, preoperative spinal stenosis (**A**,**A1**) with increased spinal canal area on postoperative day one MRI (**B**,**B1**). There was modest restenosis that occurred at postoperative 6 months (**C**,**C1**), which improved modestly at postoperative one year with overall improved spinal canal area (**D**,**D1**).

In type C (progressive expansion): SCA expanded on postoperative day one. Postoperative 6 months SCA measured ≤30% decrease in SCA expanded state compared to postoperative day one. Postoperative 1 year SCA improved from postoperative 6 months to within 10% of postoperative day one SCA (Figure 6). 

**Figure 7:**
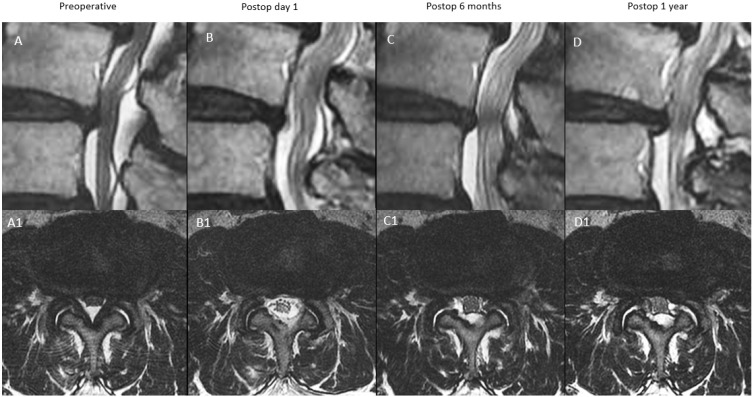
MRI appearance of remodeling patterns at preoperative, postoperative day 1, postoperative 6 months and postoperative 1 year with mid-sagittal cut in the top row and corresponding axial cut in the bottom row. Type D: remodeling type, preoperative spinal stenosis (**A**,**A1**) with increased spinal canal area on postoperative day one MRI (**B**,**B1**). There was moderate restenosis that occurred at postoperative 6 months (**C**,**C1**), which improved modestly at postoperative one year with overall still significant moderate stenosis of the spinal canal (**D**,**D1**).

In type D (restenosis): SCA expanded on postoperative day one but decreased in SCA > 30% was observed at postoperative 6 months. There was an increment in SCA at postoperative 1 year compared to postoperative 6 months; however, overall, it remained decreased >30% of postoperative day one SCA at one year (Figure 7). 

All the measurements were taken on T2-weighted axial images parallel to the disc space at the level of surgery using an INFINITT PACS M6 Version (INFINITT Healthcare Corporation, Seoul, Republic of Korea) (Table 1).

There were 32 type A, 53 type B, 30 type C and 11 type D remodeling patterns observed in the 126 levels of decompression. The basic biodata and involved levels were shown in Table 2. 

There was a statistically significant difference in age, with type B and D being significantly older than type A and D. The mean follow-up was 27.55 (17–38) months. The complication rate was significantly higher in the type D restenosis cohort (45.5%) compared to type A (0%), B (13.2%) and C (10%) cohorts, *p* < 0.05. In terms of complication management, there was no complication in the type A cohort. In the type B cohort, there were three dura tears, which were treated with the patch blocking repair technique without sequelae [19]; three facet cysts were treated conservatively, and there was one case of instability, which required endoscopic transforaminal lumbar interbody fusion (ETLIF) one year after index surgery [20,21,22]. In the type C cohort, there were two cases of dura tear, which were repaired with patch blocking technique without sequelae and one case of instability, which required ETLIF one year after index surgery. In the type D cohort, there were two cases of dura tear, which were repaired with patch blocking technique without sequelae and two cases of instability that required ETLIF, with one case at postoperative one year, and the other case at postoperative two years (Table 2).

The type B and type D cohorts had significantly lower axial spinal canal area with 76.40 ± 24.58 mm_2_ and 65.47 ± 17.98 mm_2,_ respectively, compared to type A and C with 97.39 ± 41.26 mm_2_ and 95.18 ± 38.71 mm_2_, respectively, *p* < 0.05. There was a corresponding significant difference between the postoperative higher axial spinal canal area in type A and C and type B and D in the upper end plate, mid disc region but no significant difference in the lower end plate region at postoperative day one, 6 months and one year (Table 2). 

There were six cases of revision surgery within 12 months (4.76%) in four cohorts. Overall, four of the six cases required revision endoscopic fusion, one case of transforaminal endoscopic lumbar discectomy and one case of revision LE-ULBD. There was no clinically significant difference among the four subgroups in terms of VAS and ODI and the percentage of good to excellent outcomes (Table 2).

In subtype analysis of data, within each subtype of A-D, there is a statistically significant improvement in VAS and ODI at postoperative 1 week, 3 months and final follow-up. Despite differences in varying degrees of restenosis, overall, there is a statistically significant increment in the spinal canal area in the upper end plate, mid disc and lower end plate at postoperative day 1, 6 months and 1 year. 

### 3.2. Combined Clinical and Radiographic Parameters of Patients Who Underwent LE-ULBD with 4 Subtypes of Postoperative MRI Canal Remodeling

Overall, there was a statistically significant improvement in terms of VAS, ODI and increment in the postoperative axial cut spinal canal area at postoperative day one, six months and one year MRI compared to the preoperative state. Both VAS and ODI at all time points meet the MCID of VAS (2.5–3.5) and ODI (15–16.5) [23] (Table 3).

### 3.3. Comparison of Clinical and Radiographic Parameters of Patients Who Underwent LE-ULBD with Four Subtypes of Postoperative MRI Canal Remodeling

We performed subgroup analysis of the four types of remodeling pattern in LE-ULBD. We found that each subtype managed to achieve MCID in VAS and ODI and there was no statistically significant difference among the four subtypes in clinical improvement. 

In terms of MRI axial cut analysis, there is a statistically significant difference in the spinal canal area axial cut increment at postoperative day one, with type B, C and D having more increment than type A in the upper end plate, mid disc and lower endplate. Type B and C maintained a higher spinal canal area increment than type A and D in the upper end plate and mid disc at postoperative six months and one year, *p* < 0.05. At the lower endplate, type D had a significantly higher spinal canal area increment than type B and C, which were higher than type A, *p* < 0.05 (Table 4).

## 4. Discussion

The benefits of lumbar endoscopic spine surgery in the treatment of degenerative spine conditions have been well described [1,3]. Studies showed shorter hospital admission, less blood loss, less soft tissue damage and a lower infection rate compared to open decompression. Some recent studies suggested the trend of fewer complications in endoscopic surgical decompression [12,24].

A biomechanical study showed endoscopic unilateral laminotomy with bilateral decompression has less biomechanical disturbance than medial facetectomy through open technique [25]. Clinical results of LE-ULBD demonstrated efficacy in short to medium- term follow-up [8,9,12,26,27]. In the limited literature on the cross-sectional area increment in the evaluation of radiographic efficacy of LE-ULBD, a statistically significant increment in spinal canal parameters was demonstrated [10,28,29]. However, there is no literature demonstrating the progress of remodeling of the spinal canal in repeated MRI at 6 months and one year and clinical correlation with the remodeling process. 

In our series, we found there are four types of remodeling in the spinal canal area after an adequate decompression was achieved on postoperative day one of LE-ULBD, namely, type A (continuous expanded), B (remodeling with restenosis at 6 months and delayed expansion at one year), C (progressive expansion) and D (restenosis). 

**Figure 8:**
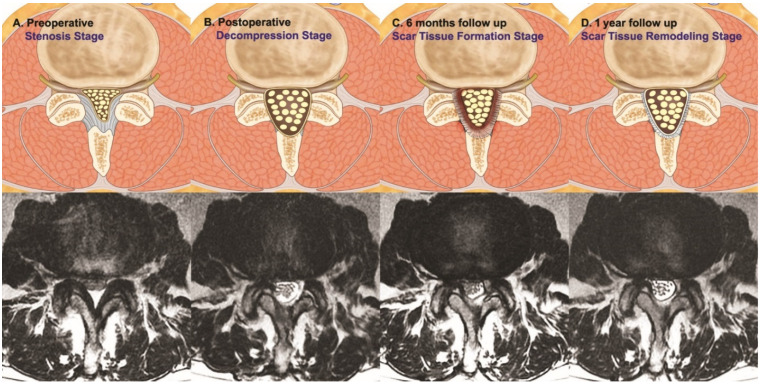
Illustration of the pattern of proposed stages of remodeling and underlying mechanism. The preoperative stenosis stage was relieved by lumbar endoscopic unilateral laminotomy with bilateral decompression to the postoperative decompressed stage. Scar tissue formation at postoperative 6 months led to restenosis at scar tissue formation stage. Further remodeling of the scar tissue was found in postoperative one year follow-up MRI scan.

We postulated the pathophysiological process of remodeling is due to the formation of granulation tissue and subsequent scar formation, which leads to asymptomatic restenosis as demonstrated by decreasing SCA. The scar which was formed in the spinal canal was remodeled in different degrees, hence giving variation in the four types of remodeling pattern in our series (Figure 8). 

**Figure 9:**
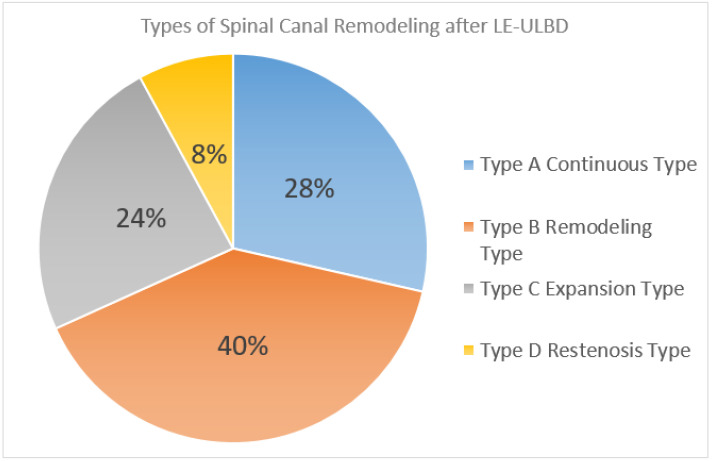
Distribution pie chart of the various types of spinal canal remodeling pattern after lumbar endoscopic unilateral laminotomy with bilateral decompression (LE-ULBD). Type A: continuous expanded spinal canal, type B: restenosis with delayed expansion, type C: progressive expansion and type D: restenosis.

The most common type of remodeling pattern was type B (40%), followed by type A (28%), C (24%) and D (8%) (Figure 9). 

Despite having four different types of remodeling, there were no statistically significant differences in clinical outcomes between the four groups. In particular, in the type D (restenosis) group, there was an interesting finding that despite less expansion in the SCA, there was a statistically significant improvement in all clinical parameters. This demonstrated that despite less increment in the spinal canal area, there was sufficient decompression to maintain good clinical outcomes in this cohort of patients. This observation echoed the findings in the literature that moderate stenosis in the spinal canal is sufficient for patients to be asymptomatic [30]. There is also a suggestion that there is no straightforward association of stenosis of the dural sac with patient symptoms and functional capacity [31]. We found factors that lead to a statistically significant increase in the risk of remodeling with delayed expansion and restenosis: (1) age, (2) preoperative MRI with more severe stenosis in the upper end plate, mid disc and lower end plate, (3) complications. The mean ages of type B and D cases are 7 years older than type A and C (*p* < 0.025). There is a significantly tighter spinal canal area in the upper endplate, mid-disc and lower endplate in type B (77.88 ± 4.70, 56.33 ± 4.63, 70.63 ± 5.05) and type D (64.29 ± 10.51, 42.90 ± 10.34, 62.93 ± 11.29) as compared to type A (93.03 ± 5.54, 80.55 ± 5.45, 94.67 ± 5.95) and C (95.18 ± 6.07, 84.00 ± 5.97, 92.68 ± 6.52), respectively, *p* < 0.05. There is a higher complication rate in the type D restenosis group compared to the other three types. We postulated that postoperative complication is a risk factor for restenosis. The authors hypothesized that instability is a cause of remodeling and restenosis, though we found one case in each of type B and C and two cases in type D remodeling. There are a small number of cases in each subgroup allowing exploration of the causal link between instability and restenosis. There were four cases (3.17%) with worsening of instability of which two cases (2.38%) required revision endoscopic posterolateral transforaminal interbody fusion [32,33,34]. Overall, despite the fact that there is some modest to moderate form of restenosis in the spinal canal area in our cohort of patients, there is no significant correlation to clinical outcomes. 

Overall, this study highlights the issue of careful interpretation of postoperative spine MRI with correlation to clinical symptoms. The literature is divided in the adequacy of spinal decompression in uniportal LE-ULBD in comparison to other minimally invasive approaches such as unilateral biportal endoscopic decompression (UBE) and microscopic tubular lumbar decompression. This is further confounded by the steep learning curve in uniportal lumbar endoscopic decompression [35,36,37]. Heo et al. compared the three subtypes of decompression, showing that LE-ULBD had the least dural expansion compared to UBE and tubular surgeries despite no clinical difference in outcomes [38]. Lee et al. showed similar radiographic and clinical outcomes in LE-ULBD, tubular and open microscopic groups [12]. Interestingly, Carrascosa-Granada showed in their small sample cohort study that endoscopic decompression has a larger dural sac expansion compared to tubular decompression without statistical significance [39]. In our cohort, there is a statistically significant dura sac expansion in all subtypes of remodeling groups with good clinical outcomes correlation. 

As LE-ULBD is one of the more advanced and newer technical developments in uniportal full endoscopic surgery, long-term data are lacking. Some of the literature in transforaminal endoscopic surgery for lumbar disc herniations shows no significant difference in long term outcomes in leg pain reduction, overall improvement and complication rate

## 5. Limitations

There are several differences and possible confounding factors in this study: the data were obtained as a retrospective single cohort study under one senior surgeon with a low number of patients; there could be inherent selection and performance bias in the study. One year of follow-up is relatively short to understand the long-term prognosis of this cohort of patients. A control cohort of patients who underwent open posterior lumbar decompression would improve the quality of the study. Additionally, of 213 patients who underwent single-level LE-ULBD only 126 patients completed the postoperative one year MRI. As postoperative MRI is voluntary, the lost-to-follow-up study is inherent in this format of study, and it is a possible confounder. As the authors in this study performed nearly all cases of single-level stenosis with an endoscope, we limited the selection bias. Pre-operative data such as comorbidities, Charlson Morrison Index, BMI and smoking history were not collected which might introduce confounders in the study. We limited these confounding factors by having the same team of anesthetists and surgeons for both cohorts of operations performed in the data set for both groups. The follow-up was of medium-term duration and we continued to follow-up on these patients with a view to showing the effect of a longer follow-up in the future to evaluate the clinical and radiological data in the long term. 

## 6. Conclusions

After full endoscopic lumbar decompression, despite achieving sufficient decompression immediately postoperatively, varying severity of asymptomatic restenosis was found in the postoperative six months MRI without clinical significance. Further remodeling with a varying degree of increment of spinal canal area occurs at postoperative one year with overall good clinical outcomes.

## Figures and Tables

**Table 1:** Description of the types of remodeling patterns and corresponding spinal canal area measurement at postoperative day one, 6 months and one year.

Type	Description	Postoperative Day 1 Spinal Canal Area mm^2^	Postoperative 6 Months Spinal Canal Area mm^2^	Postoperative 1 Year Spinal Canal Area mm^2^
A: Continuous Type	There is significant decompression with increased spinal canal area on postoperative day one, a modest drop at postoperative 6 months and close to postoperative 6 months value at postoperative one year	X	≥70% X	70–90% X (close to postoperative six months value)
B: Remodelling Type	There is significant decompression with increased spinal canal area on postoperative day one, a significant drop at postoperative 6 months and modest improvement at one year but less than 90% of the postoperative day one spinal canal area	X	<70% X	70–90% X
C: Expansion Type	There is significant decompression with increased spinal canal area on postoperative day one, a modest drop at postoperative 6 months and significant improvement at one year with more than 90% of the postoperative day one spinal canal area	X	≥70% X	90–100% X
D: Restenosis Type	There is significant decompression with increased spinal canal area on postoperative day one, a significant drop at postoperative 6 months and minimal improvement at one year with <70% of the postoperative day one spinal canal area	X	<70% X	<70% X

**Table 2:** Baseline demographics and characteristics of the four types of remodeling after lumbar endoscopic unilateral laminotomy with bilateral decompression (LE-ULBD). The chi-square test for categorical variable and ANOVA test for continuous variables were used to compare the groups. Tukey’s multiple comparison test was used for the post-hoc test in continuous variables.

	Type A	Type B	Type C	Type D	Combined	*p* Value
Number of patients	32	53	30	11	126	N/A
Number of Patients with Level Lumbar Two Three	2	5	2	3	12	N/A
Number of Patients with Level Lumbar Three Four	6	14	9	3	32	N/A
Number of Patients with Level Lumbar Four Five	13	30	14	3	60	N/A
Number of Patients with Level Lumbar Five Sacral One	11	3	4	2	20	N/A
Age (mean, range in years)	59.41 (21–80)	67.60 (28–83)	60.33 (21–86)	67.18 (57–78)	63.75 (21–86)	0.007
F/U Period (mean, range in years)	26.53 (17–37)	28.13 (18–36)	26.77 (17–35)	29.81 (26–38)	27.55 (17–38)	0.184
Male:Female Ratio	10:22	28:25	12:18	5:6	55:71	0.262
Complication Rate	0.00	13.21	10.00	45.45	11.90	0.001
Revision Surgery	2	1	1	2	6	0.219
Preoperative MRI Measurement Area in Upper End Plate (mean, SD) mm^2^	97.39 ± 41.26	76.40 ± 24.58	95.18 ± 38.71	65.47 ± 17.98	85.25 ± 34.29	0.003
Postoperative Day 1 MRI Measurement in Upper End Plate (mean, SD) mm^2^	136.55 ± 47.18	123.82 ± 24.79	146.63 ± 48.31	114.57 ± 21.10	131.68 ± 38.68	0.023
Postoperative 6 months MRI Measurement Area in Upper End Plate (mean, SD) mm^2^	126.33 ± 48.14	108.46 ± 23.30	135.78 ± 45.70	92.77 ± 17.03	118.14 ± 38.69	<0.001
Postoperative One Year In Upper End Plate MRI Measurement Area (mean, SD) mm^2^	125.38 ± 39.54	118.79 ± 24.77	144.67 ± 47.42	103.64 ± 14.75	125.30 ± 36.49	0.002
Preoperative MRI Measurement Area in Mid Disc (mean, SD) mm^2^	82.36 ± 35.18	56.82 ± 19.57	84.00 ± 48.86	45.31 ± 17.04	68.77 ± 35.43	<0.001
Postoperative Day 1 MRI Measurement in Mid Disc (mean, SD) mm^2^	137.52 ± 46.68	129.65 ± 25.80	150.85 ± 53.20	116.96 ± 24.01	135.59 ± 40.28	0.047
Postoperative 6 months MRI Measurement Area in Mid Disc (mean, SD) mm^2^	129.59 ± 43.70	106.61 ± 24.99	138.92 ± 50.36	86.86 ± 22.64	115.88 ± 40.05	<0.001
Postoperative One Year in Mid Disc (mean, SD) mm^2^	127.21 ± 44.98	119.76 ± 27.53	148.33 ± 54.54	95.15 ± 21.74	126.31 ± 42.01	0.001
Preoperative MRI Measurement Area in Lower Endplate (mean, SD) mm^2^	93.72 ± 37.69	72.65 ± 30.87	92.68 ± 44.50	65.43 ± 28.73	82.14 ± 37.39	0.010
Postoperative Day 1 MRI Measurement in Lower Endplate (mean, SD) mm^2^	143.61 ± 46.06	136.28 ± 31.51	151.18 ± 51.79	146.86 ± 37.31	142.61 ± 41.35	0.451
Postoperative 6 months MRI Measurement Area in Lower Endplate (mean, SD) mm^2^	134.52 ± 44.08	125.41 ± 28.61	146.6 ± 50.37	125.13 ± 36.53	132.74 ± 39.91	0.117
Postoperative One Year in Lower Endplate (mean, SD) mm^2^	134.76 ± 45.27	127.36 ± 32.34	148.00 ± 49.34	123.83 ± 33.56	133.85 ± 40.90	0.132
Preoperative VAS (mean, SD)	7.66 ± 1.18	7.74 ± 1.35	7.30 ± 1.49	7.73 ± 1.19	7.61 ± 1.33	0.529
Postoperative VAS at 1 week(mean, SD)	3.00 ± 0.51	3.11 ± 0.51	3.10 ± 0.76	3.09 ± 0.54	3.08 ± 0.57	0.844
Postoperative VAS at 3 months(mean, SD)	2.06 ± 0.80	2.15 ± 0.84	2.17 ± 0.87	2.45 ± 0.82	2.39 ± 0.78	0.899
Postoperative VAS at final follow-up(mean, SD)	134.76 ± 45.27	127.36 ± 32.34	148.00 ± 49.34	123.83 ± 33.56	2.16 ± 0.83	0.616
Preoperative ODI(mean, SD)	73.75 ± 8.62	74.57 ± 9.58	70.93 ± 9.79	74.73 ± 7.55	73.51 ± 9.25	0.361
Postoperative ODI at 1 week(mean, SD)	30.31 ± 4.22	30.38 ± 4.42	31.60 ± 7.11	31.27 ± 5.00	30.73 ± 5.16	0.703
Postoperative ODI at 3 months (mean, SD)	26.88 ± 5.28	26.42 ± 4.66	27.07 ± 6.53	26.55 ± 4.30	26.70 ± 5.24	0.952
Postoperative ODI at final follow-up(mean, SD)	24.56 ± 4.85	24.57 ± 5.09	25.33 ± 6.31	25.45 ± 4.66	24.83 ± 5.27	0.889
Percentage MacNab Good To Excellent Outcome(%)	96.88	96.23	96.67	90.91	96.03	0.837

**Table 3:** Combined Clinical and Radiographic Parameters of Patients who underwent LE-ULBD with Four Types of Postoperative MRI canal remodeling. *p*-Value was derived from paired *t*-test.

Combined Data LE-ULBD Type A to D	Mean	Std. Deviation	*p* Value
VAS improvement at 1 weeks	4.53	1.35	<0.001
VAS improvement at 3 months	5.22	1.53	<0.001
VAS improvement at final follow-up	5.45	1.67	<0.001
ODI improvement at 1 weeks	42.78	10.00	<0.001
ODI improvement at 3 months	46.81	10.25	<0.001
ODI improvement at final follow-up	48.68	10.80	<0.001
Increment of day 1 postoperative MRI spinal canal in upper end plate (mean, SD) mm^2^	46.43	20.35	<0.001
Increment of 6 months postoperative MRI spinal canal area in upper end plate (mean, SD) mm^2^	32.89	19.75	<0.001
Increment of one year postoperative MRI spinal canal area in upper end plate (mean, SD) mm^2^	40.05	27.80	<0.001
Increment of day 1 postoperative MRI spinal canal area in mid disc (mean, SD) mm^2^	66.82	27.85	<0.001
Increment of 6 months postoperative MRI spinal canal area in mid disc (mean, SD) mm^2^	47.10	25.37	<0.001
Increment of one year postoperative MRI spinal canal area in mid disc (mean, SD) mm^2^	57.53	27.04	<0.001
Increment of day 1 postoperative MRI spinal canal area in lower end plate (mean, SD) mm^2^	60.47	27.58	<0.001
Increment of 6 months postoperative MRI spinal canal area in lower end plate (mean, SD) mm^2^	50.61	24.64	<0.001
Increment of one year postoperative MRI spinal canal area in lower end plate (mean, SD) mm^2^	51.71	25.12	<0.001

**Table 4:** Comparison of Clinical and Radiographic Parameters of Patients who underwent LE-ULBD with Four Types of Postoperative MRI canal remodeling. *p*-Value was derived from ANOVA test, and Tukey’s multiple comparison test was used for the post-hoc test.

Group Charateristics	Type A	Type B	Type C	Type D	*p* Value
Improvement of VAS at 1 week	4.66 ± 1.26	4.62 ± 1.40	4.20 ± 1.27	4.64 ± 1.57	0.499
Improvement of VAS at 3 months	5.28 ± 1.37	5.40 ± 1.61	4.83 ± 1.56	5.27 ± 1.49	0.446
Improvement of VAS at final FU	5.59 ± 1.58	5.58 ± 1.71	5.13 ± 1.72	5.27 ± 1.68	0.624
Improvement of ODI at 1 week	43.44 ± 9.16	44.19 ± 10.36	39.33 ± 9.21	43.45 ± 11.80	0.187
Improvement of ODI at 3 months	46.88 ± 9.85	48.15 ± 10.83	43.87 ± 9.92	48.18 ± 8.92	0.313
Improvement of ODI at final FU	49.19 ± 10.51	50.00 ± 11.29	45.60 ± 10.58	49.27 ± 9.39	0.346
Increment of day 1 postoperative MRI spinal canal in upper end plate (mean, SD) mm^2^	39.16 ± 22.73	47.42 ± 18.77	51.45 ± 18.16	49.10 ± 23.05	0.010
Increment of 6 months postoperative MRI spinal canal area in upper end plate (mean, SD) mm^2^	28.95 ± 22.14	32.06 ± 19.40	40.60 ± 16.93	27.30 ± 17.43	0.076
Increment of one year postoperative MRI spinal canal area in upper end plate (mean, SD) mm^2^	28.00 ± 42.57	42.38 ± 19.29	49.49 ± 18.41	38.18 ± 18.94	0.018
Increment of day 1 postoperative MRI spinal canal area in mid disc (mean, SD) mm^2^	55.16 ± 27.51	72.83 ± 23.87	66.85 ± 34.48	71.65 ± 16.87	0.036
Increment of 6 months postoperative MRI spinal canal area in mid disc (mean, SD) mm^2^	47.23 ± 25.88	49.79 ± 21.93	54.92 ± 30.70	41.55 ± 12.92	0.030
Increment of one year postoperative MRI spinal canal area in mid disc (mean, SD) mm^2^	44.86 ± 25.73	62.94 ± 24.43	64.33 ± 31.82	49.83 ± 13.31	0.006
Increment of day 1 postoperative MRI spinal canal area in lower end plate (mean, SD) mm^2^	49.89 ± 34.50	63.63 ± 23.70	58.50 ± 24.27	81.43 ± 16.81	0.007
Increment of 6 months postoperative MRI spinal canal area in lower end plate (mean, SD) mm^2^	40.80 ± 29.35	52.76 ± 22.70	53.92 ± 22.18	59.70 ± 18.80	0.055
Increment of one year postoperative MRI spinal canal area in lower end plate (mean, SD) mm^2^	41.04 ± 28.56	54.72 ± 24.29	55.32 ± 22.49	58.40 ± 18.05	0.045

## Data Availability

The data presented in this study are available on request from the corresponding author. The data are not publicly available due to patient data protection act.
